# Generalized pole figures from post-processing whole Debye–Scherrer patterns for microstructural analysis on deformed materials

**DOI:** 10.1107/S160057752200220X

**Published:** 2022-04-26

**Authors:** Emanuel Alejandro Benatti, Natalia Soledad De Vincentis, Nowfal Al-Hamdany, Norbert Schell, Heinz-Günter Brokmeier, Martina Avalos, Raúl Eduardo Bolmaro

**Affiliations:** a Física y Micromecánica de Materiales Heterogéneos, Instituto de Física Rosario – CONICET, Bv. 27 de Febrero 210, Rosario, Santa Fe S2000EZP, Argentina; b Institute of Materials Mechanics, Department of Laser Processing and Structural Assessment, Helmholtz-Zentrum Hereon, Max-Planck Strasse 1, 21502 Geestacht, Germany; c Institut für Werkstoffkunde und Werkstofftechnik, TU Clausthal, Agricolastrasse 6, 38678 Clausthal-Zellerfeld, Germany; d GEMS Outstation, Helmholtz-Zentrum Geesthacht, Notkestrasse 85, 22607 Hamburg, Germany

**Keywords:** X-ray diffraction, generalized ODF, electron backscatter diffraction, orientation distribution function

## Abstract

Debye–Scherrer patterns, obtained from X-ray diffraction experiments using synchrotron light in transmission geometry, were analysed to construct generalized pole figures, and further used as input for an orientation distribution function inversion algorithm. By using Langford’s method for separating strain and size contributions to peak broadening, it was possible, for the first time, to obtain full domain size and dislocation density generalized distribution functions (GDFs). This method was applied to cold-rolled and annealed interstitial-free steel. The predictions made using GDFs were corroborated by electron backscatter diffraction measurements and were also consistent with what was previously known for this kind of material under these conditions.

## Abstract 2

Debye–Scherrer patterns, obtained from X-ray diffraction experiments using synchrotron light in transmission geometry, were analysed to construct generalized pole figures. Generalized orientation distribution functions of cold-rolled and then annealed interstitial-free steel were obtained to investigate microstructure developments in this material.

## 1. Introduction

### 1.1. An overview of the Langford model for line profile analysis

It is well known that thermo-mechanical processing strongly influences the development of material microstructures. Together with optical microscopy, X-ray diffraction (XRD) was one of the first tools employed to study the development of microstructures in deformed materials.

Currently, there are three main techniques for microstructure determination: transmission electron microscopy (TEM), electron backscatter diffraction in SEM microscopes (SEM-EBSD) and line profile analysis from X-ray diffraction peaks (XRD-LPA). TEM has the highest spatial resolution but the poorest statistics, whereas XRD provides an indirect method for the characterization of microstructures by meaningful interpretation of diffraction patterns. It has the worst spatial resolution, but the best statistics. SEM-EBSD resolution lies somewhere between both techniques and may help to link the sometimes inconsistent conclusions obtained from TEM and XRD.

In this work, we focus on the use of XRD-LPA to obtain information on the microstructure of materials, and the way to correlate the defect (dislocations, grain size *etc*.) storage capacity with the orientation distribution or texture. Currently, there are generally no accepted models that can accurately correlate deformation levels and defect densities. A correct quantification of the microstructure remains so far a crucial task in order to determine the effect of different mechanical processes in any material.

Since the origins of XRD, it is well known that broadening of diffraction peaks is related to different kinds of defects in a crystalline lattice, aside from instrumental peak broadening discussed later. A material with low dislocation density and spherically shaped domains will produce a diffraction pattern with a full width at half-maximum (FWHM) for each {*hkl*} reflection given by

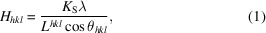

where λ is the wavelength of the incident X-ray beam, *L* ^hkl^ is the length of the coherent domain size in the *hkl* direction, θ_*hkl*_ is the Bragg angle for the *hkl* reflection, *H_hkl_* is its FWHM and *K*_S_ is the Scherrer constant (Scherrer, 1918 ▸).

If, on the other hand, one can neglect the broadening from large domain sizes, and assuming the peak broadening comes only from the storage of dislocations producing cell parameter changes by developing internal stresses, it can be shown that the quantity *H_hkl_* is given by (Keijser *et al.*, 1982 ▸)

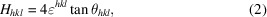

where ɛ^*hkl*^ is the mean-square strain of the crystalline lattice, observed in the *hkl* direction. In a more general case, the peak broadening for a given reflection will have contributions from both size and strain effects. By assuming that the size contribution will shape the diffraction peak as a Lorentzian distribution, and that the strain contribution will give a Gaussian shape, Langford proposed to model a diffraction peak with a Voigt function, *i.e.* the convolution of Gaussian [*G*(*x*)] and Lorentzian [*L*(*x*)] functions (Thompson *et al.*, 1987 ▸), 

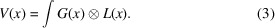

In equation (3), both the Gaussian and the Lorentzian functions have different FWHMs, *H*_G_ for the Gaussian and *H*_L_ for the Lorentzian, so once a particular peak has been fitted with a Voigt function, the values *H*_L_ and *H*_G_ can be inserted into equations (1) and (2), respectively, to estimate the crystallite size and the mean strain in a particular direction of a given sample.

In practice, it is easier to work with the pseudo-Voigt approximation,^1^ which substitutes the pair (*H*_G_, *H*_L_) with the pair (*H*, η),

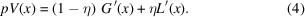

The *pV*(*x*) function is a linear combination of Lorentzian and Gaussian functions of the same FWHM *H*, with a mixing parameter η. The pseudo-Voigt function expressed in equation (4) has the advantage of being numerically easier to compute than in equation (3). The mapping between the pairs (*H*_G_, *H*_L_) and (*H*, η) can easily be obtained using the numerical approximation provided by Thompson *et al.* (1987 ▸).

This rather simple interpretation has received a correct criticism regarding the fact that neither the grain size nor the dislocation distortion can be completely described by Lorentzian and Gaussian distributions, respectively. Both are actually represented by complex distributions, only approximately described by the Voigt distribution as a convolution of perfectly separated effects.

In fact, it can be seen that if dislocations are arranged in compact arrays, the consequent broadening tends to be Lorentzian, whereas for uncorrelated dislocations the broadening is Gaussian (Kerber, 2011 ▸). This means that for compact arrays of dislocations, equation (1) can be interpreted as the average column length of the coherently diffracting crystals along the *hkl* direction. In this sense, *L* ^*hkl*^ will be determined by the minimum between the actual length of the crystallites in the *hkl* direction and the average separation between compact arrays of dislocations. On the other hand, equation (2) can be interpreted as the mean strain produced by random distributed dislocations in the material in the *hkl* direction. When interpreted this way, the values of *L^hkl^* and ɛ*^hkl^* can be used for estimating dislocation density in a material using the approach of Williamson & Smallman (1956 ▸) and Chowdhury *et al.* (2010 ▸), who developed an expression that allows estimation of dislocation densities from the *L* ^*hkl*^ and ɛ^*hkl*^ values obtained from equations (1) and (2),

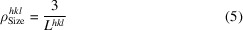




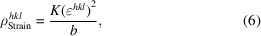

where *K* is a constant that depends on the material elastic constants and *b* is the magnitude of the Burgers vector. Once the dislocation densities from size and strain are obtained, the average dislocation density for the {*hkl*} plane family can be estimated from the geometric average of such quantities (Kapoor *et al.*, 2004 ▸),

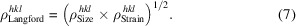

This way, the total dislocation density of a material will be between the values given by equations (2) and (6) and that obtained from taking into account the contributions given by equations (1), (2), (5), (6) and (7). The more random the array of dislocations, the more accurate the value given by equation (6), whereas, if the dislocations are stored in compact arrays, the value given by equation (7) would be preferable.

It also follows from the previous discussion that dislocation arrays composed of loosely packed dislocations can be better modelled by a complete separation of independent effects following the Langford model. In such cases dislocation distortion follows quite closely a Gaussian distribution, which can be easily separated from the domain size broadening (Mittemeijer & Scardi, 2003 ▸; Kerber, 2011 ▸). However, if dislocations are stored in compact arrays it is difficult to accurately separate size contribution from the contribution of non-random dislocations in the peak broadening.

### 1.2. Criticisms and alternative methods to Langford analysis

As stated earlier, a major problem that arises when employing Langford’s method is the assumption that broadening caused by dislocations and crystallite size cannot be properly reproduced by taking into account solely Lorentzian and Gaussian functions, which makes the method biased (Scardi *et al.*, 2004 ▸). The bias can be reduced if the whole pattern is analyzed instead of studying a single peak. Among the methods that employ this approach we may mention the methods of Williamson–Hall (WH), Warren–Averbach (WA) and all modified versions (Hall, 1949 ▸; Averbach & Warren, 1949 ▸; Ungár & Tichy, 1999 ▸; Ungár *et al.*, 1999 ▸, 2001*a* ▸,*b* ▸). These methods attempt to correct the problem that arises from performing a single peak analysis, and separates the size and strain contributions by taking into account that size broadening is order-independent, whereas strain broadening is order-dependent.

The modified versions of WH and WA methods improve the estimation of the dislocation density by taking into account the anisotropic broadening of dislocations using the so-called contrast factors (*C* ^*hkl*^). These factors take into account that the strain produced by a dislocation is dependent on the direction that the dislocation itself is observed as well as the dislocation type, *i.e.* edge or screw (Ungár & Tichy, 1999 ▸; Ungár *et al.*, 1999 ▸, 2001*a* ▸,*b* ▸; Scardi *et al.*, 2004 ▸).

The other method employed to separate size and strain contributions is to fit the Fourier coefficients of the diffraction profiles. The upside of this method is that it is completely unbiased, since it does not assume any shape for the profiles, although it also requires measuring several reflections in order to separate strain and size contributions. Fourier methods also naturally include the concept of contrast factors, and take into account the compactness of dislocation arrays. It is through the analysis of the Fourier coefficients that we demonstrated that the asymptotic behaviour of the profiles is determined by the spatial correlation of the dislocation distribution (Borbély & Groma, 2001 ▸; Groma, 1998 ▸, 2013 ▸; Kalácska *et al.*, 2017 ▸; Székely *et al.*, 2000 ▸; Wilkens, 1970 ▸) and, when dislocations are stored in compact arrays, the tails of the diffraction profiles tend to be Lorentzian; however, when the dislocation distribution is random, the tail of the profile is Gaussian (Kerber, 2011 ▸). At this point, it is worth mentioning that, even though Langford’s method uses the FWHM of the Gaussian and Lorentzian components of the profile, those values are strongly influenced by the mixing parameter η, which in turn is determined by the asymptotic behaviour of the profile.

Among the methods based on the analysis of the Fourier coefficients, the more developed versions are the convolutional multiple-whole profile (CMWP) (Ungár *et al.*, 2001 ▸; Ribárik *et al.*, 2001 ▸; Ribárik, 2008 ▸; Gábor, 2008 ▸) and the whole powder pattern modelling (WPPM) (Scardi & Leoni, 2002 ▸).

Both modified versions of WH and WA, as well as the CMWP and WPPM methods, consider the visibility of the dislocations by employing the contrast factor *C* ^*hkl*^, which can be calculated for materials with high symmetry, if the active slip planes are known and the material has no texture. When there is texture, the dislocation density can still be determined if the reflections corresponding to each texture component can be separated (Jóni *et al.*, 2013 ▸; Ungár *et al.*, 2015 ▸). The problem persisting in this approach is that the defect distribution cannot be determined in the whole orientation space, but only in the main texture components, as long as they are easily separated. Another problem presented is that, despite the incorporation, in one way or another, of the contrast factor, they depend on so many parameters adjustable by mathematical techniques that they have to rely on the assumption of isotropic storage of dislocations and no dependence of crystallite size on crystal orientation, or they have to fix the slip systems available for the material. Otherwise, the large number of parameters cannot be fitted by relying only on the redundancy of information stemming from the many evaluated peaks.

### 1.3. The method of generalized pole figures

Two decades ago the measurement and analysis of features dependent on crystal orientation was suggested in the form of generalized pole figures (GPFs; Wcislak & Bunge, 1996 ▸). The great advantage of such an approach would be that the whole resources available for pole figure (PF) analysis would be readily available for understanding anisotropy of properties, defect accumulation *etc*. The sole condition for such treatment to be correct is that the property under analysis must be a mathematical function of crystal orientation, the same way the texture itself is. Despite the fact that this simple condition is envisioned to be fulfilled by many physical properties (almost by definition), there were also some practical difficulties precluding the complete application of that concept since its proposal. The method of GPFs is usually better used in the mode of generalized inverse pole figures to represent polar properties, which are properties depending only on the direction of a preferential crystal axis or even sometimes only on the sample orientation (Ryo & Ryo, 2016 ▸; Miller *et al.*, 2005 ▸), but no complete analysis has been carried out by resourcing to the determination of generalized distribution functions (GDFs) and recalculation of the original PFs.

When the first proposition to measure some of the orientation-dependent physical properties of polycrystalline materials was made, the first position-sensitive detectors were being installed in synchrotron sources and the detection technology appeared to be mature for attempting a complete GPF approach. However, the following steps for realizing the proposals were slow and only measurements of incomplete GPFs were achieved (Perlovich *et al.*, 2015 ▸; Cruz-Gandarilla *et al.*, 2012 ▸).

The purpose of the current work is to process advanced synchrotron experimental data to perform texture-like calculations and obtain GPFs and generalized orientation distribution functions (GODF) for physically meaningful data extracted from the resulting diffraction patterns. To this end we take the approach of Rahjmohan *et al.* (1997 ▸), in which we assume that the stored defect energy of a crystal is a function of its orientation *g* [*i.e.* *E*_c_ = *E*_c_(*g*)], which implies that the measured (average) energy in the crystal direction *h*_*j*_ in the sample direction *y* is

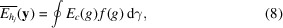

where γ corresponds to a closed path in the Euler space around the crystal direction *h*_*j*_, and *f*(*g*) is the orientation distribution function (ODF) of the material. Given that 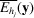 can be estimated indirectly from the broadening of the diffraction peak corresponding to the crystal direction *h*_*j*_ for many sample directions **y_i_**, it can be said that the set of 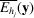 for different sample directions **y_i_** constitutes the GPF for the average defect stored energy for the crystal direction *h*_*j*_. This means that the product *E*_c_(*g*)*f*(*g*), which is the weighted stored energy for the orientation *g*, can be estimated from the energy generalized pole figures (EGPFs), 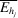 , measured for different crystal directions *h*_*j*_, using standard pole figure inversion procedures (Bunge, 1982 ▸).

In their work, Rajmohan *et al.* (1997 ▸) showed that *E*_c_ is proportional to the square of the broadening of a diffraction profile, and since the dislocation density is proportional to the square of the mean strain, equations (2) and (6) imply that it is reasonable to assume the function 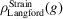 exists, and can be obtained from a treatment similar to that depicted in equation (8), substituting 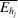 for 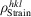 and *E*_*c*_ for 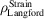 , and then applying an inversion algorithm to obtain the GDF of dislocation density weighted by the ODF, that is, 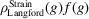 .

For this kind of analysis, the estimations of stored defect energy must be carried out by single peak analysis and, as far as we know, there is no way to perform this kind of analysis employing the methods based in WH and WA to univocally evaluate all parameters. Therefore, we approach the problem by implementing a solution according to the Langford model. The results obtained using the Langford method are later on further supported by the coherence of the results themselves and by previous knowledge of the defect storage behaviour of the current alloy. We will also validate the conclusions obtained by employing Langford’s model with EBSD measurements, and show that the conclusions obtained with the method of the GPF are consistent with those obtained independently with the EBSD measurements.

## 2. Experimental setup

Interstitial-free (IF) steel subject to cold-rolling was chosen for the experiments because it is commonly used in investigations owing to its well known texture characteristics, after both room-temperature deformation and further heat treatment. Many of the phenomena occurring during industrial and laboratory processes are well understood in these products (Ray *et al.*, 1994 ▸). A commercial-quality IF steel in the stage of ‘hot band’ (*i.e.* after high-temperature industrial reduction) was rolled to 75% reduction at room temperature, which is common for these kinds of steels. Later on, three samples were subject to heat treatments in air for 5 s at 400°C, 600°C and 730°C. At the final temperature, the usual heat treatment in cold-rolled IF steels is performed to obtain the best formability properties.

**Figure 1:**
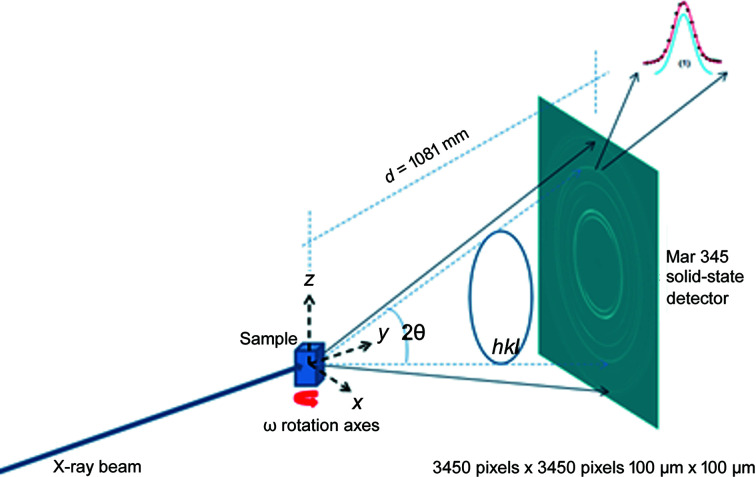
Transmission synchrotron X-ray Debye–Scherrer rings taken at HEMS at Petra III/DESY. Beam size: 100 µm × 100 µm, λ = 0.14235 Å. The Mar345 detector ensured a minimum of 20 measured points for the whole breadth for each of the measured peaks, while the Perkin–Elmer detector allowed measurement of approximately 10 points.

Diffraction experimental data were obtained at the High Energy Materials Science beamline (HEMS) at Petra III/DESY, Hamburg, Germany. Complete Debye–Scherrer rings were taken on transmission geometry with a highly parallel ∼87.1 K eV (λ = 0.14235 Å) synchrotron beam of 100 µm × 100 µm. Two types of solid-state detectors were used. First, results were taken in IF steel deformed by rolling to 75% reduction using a Mar345 detector with maximum MarMode of 3450 (effective detector size 3450 mm × 3450 mm) and highest resolution of 100 µm × 100 µm. The second round of results were obtained on heat-treated samples using the faster Perkin–Elmer (PE) detector of 2040 mm × 2040 mm with a 200 µm × 200 µm pixel size. In both sets of experiments the sample holder allowed the rotation of the sample with a 5° step (ω angle in Fig. 1 ▸). Transmission geometry was employed, and the sample was rotated from −90° to 90°, giving a collection of 37 sets of Debye–Scherrer rings for each diffraction experiment. The first setup, with 100 µm × 100 µm pixel size, guaranteed a minimum of 20 measured points for the whole breadth for each of the measured peaks. By utilizing a larger sample-to-detector distance, the larger pixel size of the PE detector was also capable of defining the peak shape with high accuracy, with each one subtending approximately 10 points. A schematic of the setup is shown in Fig. 1 ▸.

One very important compromise has to be made to ensure low instrumental peak broadening together with high statistics. Due to the very parallel synchrotron beam, compared with laboratory X-rays, sample thickness cannot be neglected as influencing instrumental line broadening. In a transmission experiment, a large sample thickness increases the peak width because the beam path tends to produce diffraction maxima at different positions on the detector. Yet a thin sample would render a small volume of crystals on the condition of having the right diffraction angle and, as a consequence, the grain statistics as well as the intensity statistics would worsen. We found that, for most materials, microstructures and textures, the best compromise is obtained with a thickness of approximately 1 mm, hence parallelepiped samples of 1 mm × 1 mm sections and 1 cm length were used.

For instrumental broadening evaluation, we also used a 1 mm-diameter LaB_6_ NIST standard sample allowing the simultaneous calculation of instrumental and sample (mostly inherent to its thickness) peak broadening. The instrumental line broadening varies between 0.020° and 0.025° for the first three Bragg reflections (110), (200) and (211), which were taken into account later on for the following line broadening investigations.

## 3. Data processing

### 3.1. XRD fitting

The standard software *FIT2D* (Hammersley *et al.*, 1996 ▸) for area frames was used to extract diffraction data (line intensity and line broadening) from the complete Debye–Scherrer rings collected. The rings were divided and integrated in 72 portions of 5° each, thus obtaining 72 diffractograms. The positions of the cakes are characterized by the γ angle in Fig. 1 ▸ and by the angle ω related to rotation position of the sample. The result is a set of 2664 diffraction patterns identified by their different (ω, γ) angles.

The experimental intensities of each diffractogram (*I*_exp_) were fitted using in-house-developed software by a sum of pseudo-Voigt functions plus a piecewise linear background function,

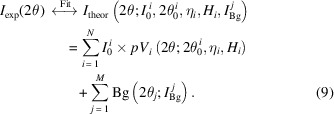

The values after the semicolon are those to be fitted by the software. The theoretical function includes a pseudo-Voigt function for each of the *N* peaks in the diffractogram, and the background function is defined as a sum of functions with *M* fixed points 2θ_*j*_. Each of these points has an associated intensity 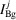 which is fitted by the software. The intensity of the background function between the *j* and *j* + 1 points is a straight line that goes from 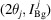 to 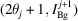 . The user can set as many background points as needed.

Once the fitting is complete, for each measured peak the user is given a set of values (*I*, 2θ_0_, *H*, η) along the sample orientation in the laboratory coordinate system (ω, γ). A coordinate transformation is then performed to take the sample axes related information to the crystal coordinate system, so the relevant quantities can be plotted as PFs (Bunge & Klein, 1996 ▸).

**Figure 2:**
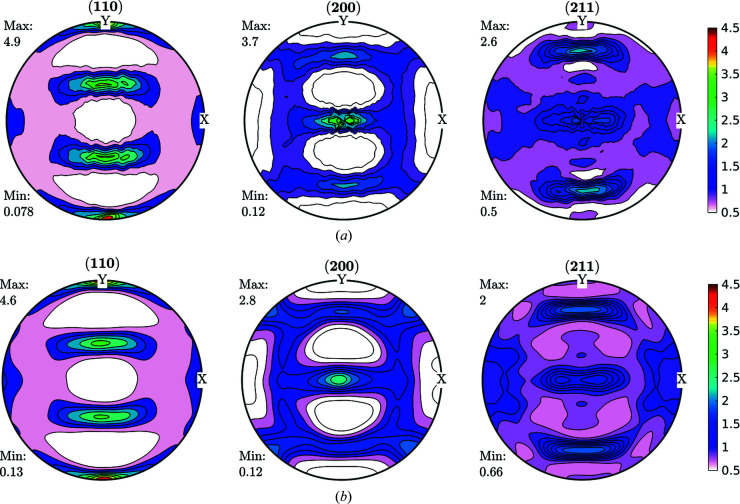
Intensity PFs for the cold-rolled sample by post-processing image plates. (*a*) Initial data obtained as described in the text. (*b*) Recalculated from ODF as coming out from *MTEX* software. The textures obtained can be easily identified as typical rolling textures for Fe.

The results were used first for calculating regular intensity pole figures, known as the texture of the material. PFs were processed by *MTEX* software (Hielscher & Schaeben, 2008 ▸) to calculate ODFs and recalculated PFs, as shown in Fig. 2 ▸, easily identified as typical rolling textures for Fe.

### 3.2. Langford analysis

As mentioned in the *Introduction* [Sec sec1], we will analyze the microstructure by employing Langford’s method. Although this method is biased, and is somehow more primitive than the other methods described in this paper, it is the only one that allows the separation of the size and strain contributions from a single peak analysis, since all the other available methods analyze the full diffraction pattern, or at least need two reflections from the same plane, to successfully separate strain and size contributions. Besides, there is no clear separation between compact dislocation arrays, which would result in the creation of subgrain boundaries, and loose arrays that are detected by X-ray peak broadening as Gaussian peaks. A rather continuous distribution of ‘compactness’ might present different limits of detection for that technique, and the mix-up between Lorentzian and Gaussian, depending mainly on X-ray beam brilliance. There is a non-proven chance of having sudden collapses of more or less loose dislocations into compact arrays, which would define a more abrupt separation between Lorentzian and Gaussian behaviour along the process.

For each *hkl* peak and sample position *i*, a pair 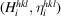 is obtained, and for each of these values the Gaussian and Lorentzian contributions to line broadening are calculated (Thompson *et al.*, 1987 ▸). With the new pair 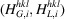 and equations (1), (2), (5) and (7), GPFs of dislocation density are obtained. We then work with the assumption presented in the *Introduction* [Sec sec1], and model the dislocation density as a function of the crystal orientation, from where it follows that the GPF can be processed using the same inversion method used for the regular PFs. The result of this process is a distribution that is actually the product of the GDF of dislocation density per crystal weighted by the ODF, as expressed in equation (8). This means that the function obtained from the inversion algorithm is the total dislocation density for the crystal orientation *g*.

Although the analysis from Rajmohan can only be reasonably applied to strain-broadening and dislocation density, we also heuristically apply the same concept with the *ad hoc* assumption that there is also a size distribution density that can be obtained by inverting a GPF of crystallite size. As Kallend & Huang (1984 ▸) already pointed out, one consequence of this assumption is that the symmetry of both size and dislocation density GPFs should be determined by the crystal symmetry. This constitutes the first conditions that should be met by all GPFs to be considered correctly analyzed and recalculated by usual PF analysis formalisms.

Apart of the symmetry of the GPFs, we also check the scope of the model by analyzing the consistency of the GPFs obtained by the inversion algorithm with the experimental ones. Finally we also compared the conclusions obtained from the GPFs with those obtained independently by EBSD measurements.

It is difficult to rationalize that a quantity such as ‘size’, which very often does not constitute a physical variable, can be considered as such in the current case. Actually the initial grain boundaries and the newly generated ones constitute a mechanism for accumulating energy, as do the dislocation arrays. The new domain boundaries are in fact a direct consequence of the accumulation of dislocations in such an amount they become indistinguishable from the original grain boundary surfaces, which are less than 1% of the final ones for large deformations, for which some authors suggest the calculation of accumulated energy as dislocation densities, see equation (5). We will attempt to describe the complete shape of a domain, surrounded by compact arrays of undiscernible dislocations, which might eventually be described by a second-rank tensor with the main axes referred to as the crystallographic axes or the sample axes.

## 4. Results

### 4.1. Quality of the fitting

Fitting was performed by following a Levenberg–Marquardt implementation of a fitting sub-routine according to equations (4) and (5). Reliable starting parameters were obtained for use as seed parameters, after a careful fit performed for a single starting diffractogram. The input parameters obtained from such initial fitting were used for each diffractogram as starting parameters.

Some critics might raise concerns about the fact that, whenever the peak intensity is low, where there are fewer grains on the proper diffraction condition, the tails of the peaks may become confused with the background and the peaks could appear wider. The fitting was performed by fixing a limit for the square root rule of the difference below which the fit was considered incorrect. Those values were not taken into account for further processing by *MTEX*, which offers the advantage of being able to calculate ODFs even though the absence of data is not confined to regularly distributed regions of the orientation space. For the measurements used in this work, the relative peak intensity background for which a good fitting was achieved was 1.33, *i.e.* if the peak is about 30% higher than the background, the quality of the fitting is good enough.

**Figure 3:**
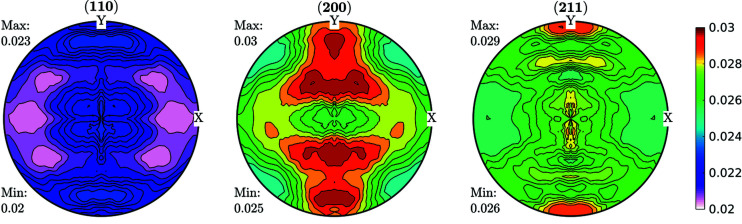
FWHM GPFs (°) for a cold-rolled IF sample. These GPFs are clearly correlated with the intensity PFs shown in Fig. 2 ▸.

**Figure 4:**
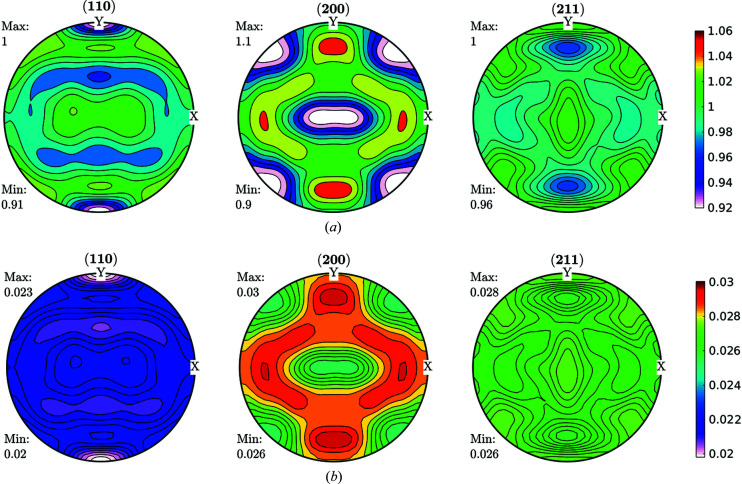
Recalculated GPF for the FWHM shown in Fig. 3 ▸, (*a*) normalized, (*b*) divided by the normalization factor (°) to obtain a real FWHM versus PF orientation. The consistencies, both qualitative and quantitative, observed between the measured and reconstructed GPFs were used as a rule of thumb criteria for checking the viability to represent the FWHM as a crystalline orientation-dependent property.

Data obtained fulfilling the convergence condition were used for further ODF function calculations and are shown in Fig. 3 ▸ for the FWHM case. As can be seen in Fig. 3 ▸, line broadening coming from the microstructure varies between 0.02° and 0.03°. These data were used as input for an ODF-PF inversion routine, and the consistency between experimental and recalculated data was used as ‘rule of thumb’ criteria for checking if it was possible to represent the FWHM as a crystalline orientation-dependent property. The recalculated FWHM GPFs are shown in Fig. 4 ▸. In Fig. 4 ▸(*a*) the GPFs were obtained from the *MTEX* inversion algorithm, which normalizes the input PFs in order to fulfil the normalization conditions of a distribution [*e.g.* Bunge (1982 ▸)]. From the *MTEX* algorithm, a normalization factor is obtained for every value of the GPF. In order to recover the original intensities, one must divide the normalized PF for such a factor, which produces the result shown in Fig. 4 ▸(*b*). In this sense, GPFs are different from intensity PFs, since normalized intensity PFs show enough information while GPFs are normalized for calculation purposes only and the correct value has to be recovered.

By comparing Figs. 3 ▸ and 4 ▸(*b*) we observe a small change in amplitude between maxima and minima in the (200) and (211) GPFs. However, both the measured and the recalculated GPFs are quite complementary with the intensity PFs, *i.e.* show their lowest values where the PFs are higher, and show their highest values where the PFs have lower intensities (see Fig. 2 ▸). There are, however, some differences between the measured and recalculated GPFs, despite which it can be said that the recalculated GPFs are reasonably consistent with the original values, which in turn highlights the consistency of the data and reproducibility of the results. Using colours in the same scale as the current figures allows us to effectively identify the incidence on the FWHM of accumulated defects on crystals characterized mainly by the orientation of particular directions with respect to the sample (PF). However, this may preclude properly separating the limits between maxima and minima.

**Figure 5:**
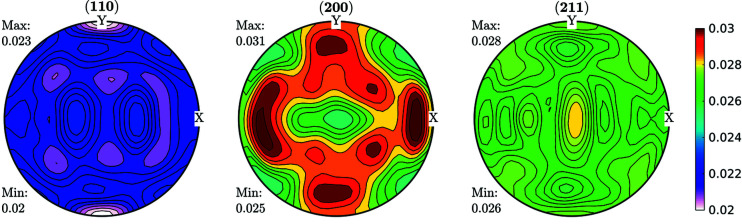
Recalculated GPFs by removal of 30% of the FWHM values corresponding to the peaks with the lowest intensities (°) for the cold-rolled sample. The stability of the GPF reconstruction was also used as criteria for studying the compatibility of the GPFs. Comparison with the results from Fig. 4 ▸ shows that the GPF reconstruction is not greatly affected by removing the data obtained from the peaks with the lowest intensities.

Another way to check the reliability of the ODF inversion from FWHM GPFs was to remove the data coming from low-intensity peaks, which provide less reliable information, and compare the results with those obtained from the complete GPFs. As illustrated in Fig. 5 ▸, the changes introduced by removing data do not affect the quality of the plots or the extremes of the recalculated GPFs, demonstrating not only the compatibility of the GPFs but also the stability of the solutions.

**Figure 6:**
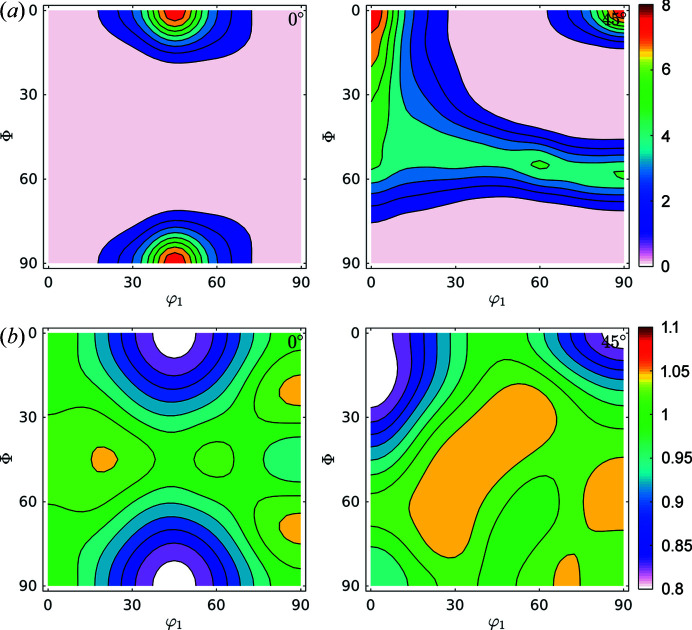
Sections at φ_2_ = 0° and φ_2_ = 45° for (*a*) ODF and (*b*) FWHM GDF for the IF cold-rolled sample. The increase of the FWHM in the orientations of the γ-fibre is in agreement with previous studies.

Fig. 6 ▸ shows the ODF and FWHM GDF (φ_2_ = 0° and φ_2_ = 45°). The ODF sections show characteristic orientations for the IF steel rolled at room temperature (Ray *et al.*, 1994 ▸). Also, the FWHM shows maxima concentrated in orientations coincident with the position of the γ-fibre at φ_2_ = 45° and minima for the ODF maxima shown at φ_2_ = 0° (Φ = 0°, φ_1_ = 45° and symmetry at Φ = 90°, φ_1_ = 45°) and at φ_2_ = 45°, Φ = 0°, φ_1_ = 0°/45°. This different storage capacity for defects during deformation of steel is in agreement with previous studies performed in the field and with the behaviour of these alloys when subject to thermal treatment.

### 4.2. Langford analysis

**Figure 7:**
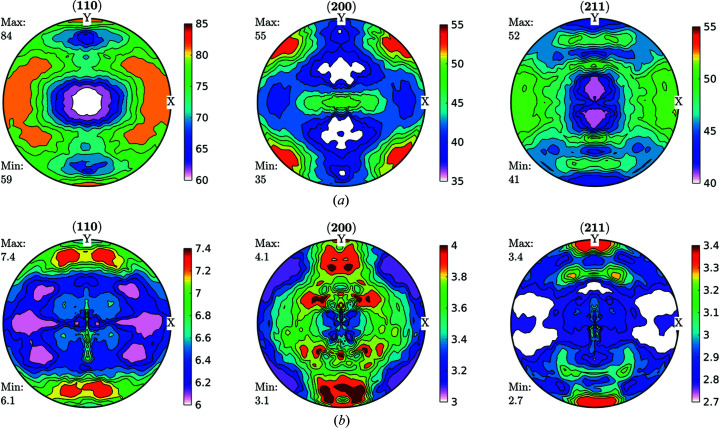
GPFs for (*a*) domain size (nm) and (*b*) dislocation density (×10^14^ m^−2^), as separated by following the Langford model from FWHM GPFs for the IF cold-rolled sample.

As explained in the *Introduction* [Sec sec1], a more physically meaningful analysis can be performed by separating FWHM GPFs into two parts by following the previously described Langford model. Domain size GPFs and strain GPFs can be calculated from equations (1), (2) and (6) and the measured broadening from the diffraction peaks, and then fed into the same ODF analysis software, accounting for the previous caveats. Original data for domain size and strain are shown in Fig. 7 ▸.

**Figure 8:**
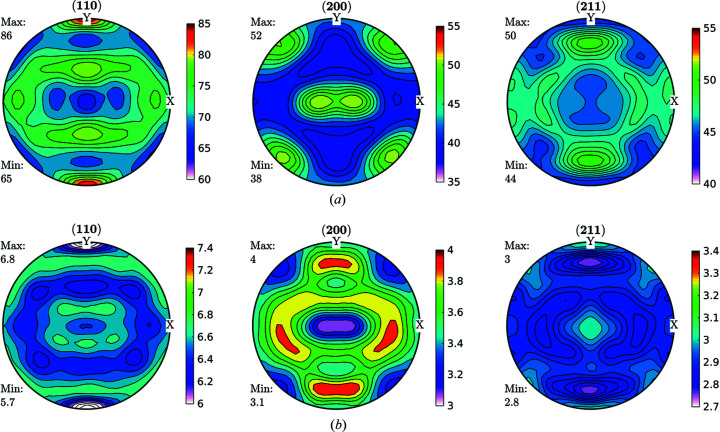
Recalculated GPFs for (*a*) domain size (nm) and (*b*) dislocation density (×10^14^ m^−2^), as separated by following the Langford model from FWHM GPFs for the IF cold-rolled sample. As well as for FWHM GPFs, we can see great consistency between recalculated and measured GPFs.

By taking the split results through the same formalism of calculating the GDFs and recalculating the GPFs, we obtained the results shown in Fig. 8 ▸.

**Figure 9:**
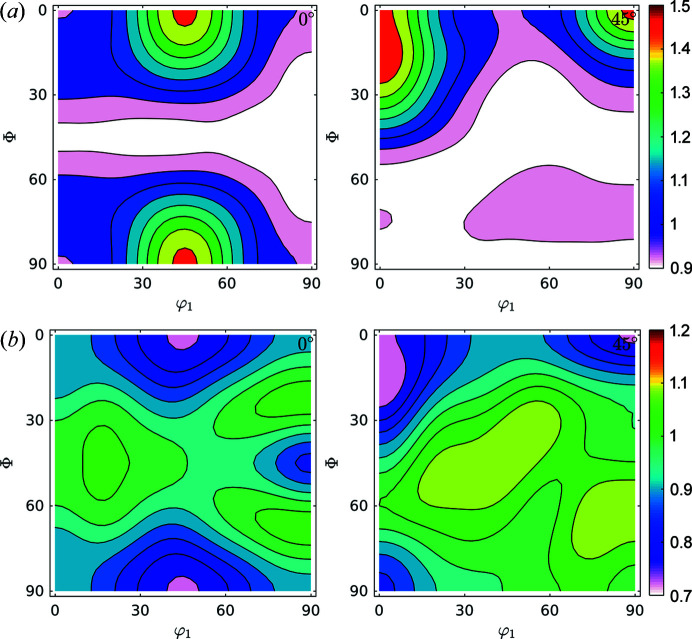
GDFs for doman size and dislocation density at φ_2_ = 0° and φ_2_ = 45° for the IF cold-rolled sample. Comparison with Fig. 6 ▸(*a*) suggests that the orientation along the γ-fibre stores more dislocations than other orientations, and that orientations in the α-fibre grew larger domains.

Fig. 9 ▸ shows the GDFs for domain sizes and dislocation densities at φ_2_ = 0° and φ_2_ = 45°. Minimum and maximum values differ by a factor of two for domain sizes and by 35% for dislocation densities. We can confirm the trend (Thomas *et al.*, 2003 ▸; Novillo *et al.*, 2003 ▸; Mohamed & Bacroix, 2000 ▸; Bacroix *et al.*, 1999 ▸; Raabe & Lücke, 1992 ▸, 1993 ▸) to store more dislocations within medium-size domains along the γ-fibre. Some larger domains, like those located in rotated cube (φ_1_, Φ, φ_2_) = (45, 0, 0)° and α(〈110〉//*RD*) components store fewer dislocations: they are larger and cleaner.

**Figure 10:**
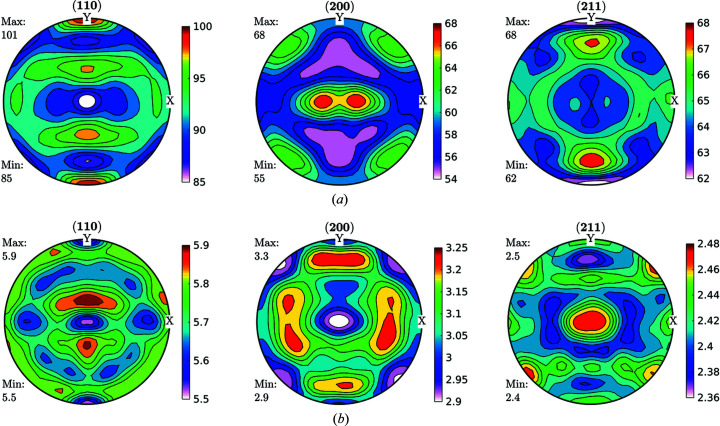
Recalculated GPFs for (*a*) domain size (nm) and (*b*) dislocation density (10^14^ m^−2^) as separated by following the Langford model from FWHM GPFs. Heat-treated sample, 5 s at 400°C.

**Figure 12:**
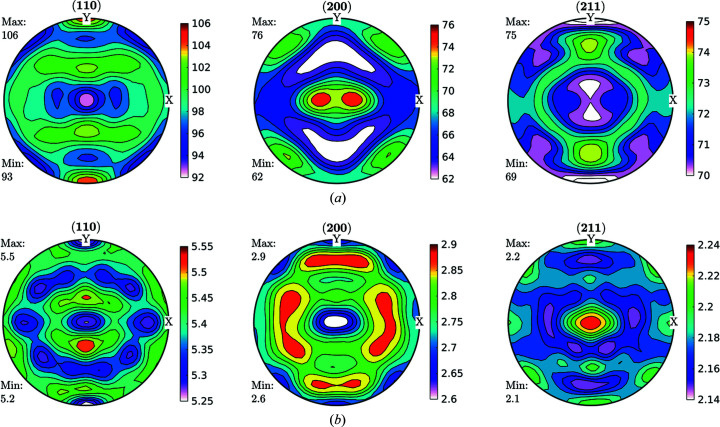
Recalculated GPFs for (*a*) domain size (nm) and (*b*) dislocation density (10^14^ m^−2^), as separated by following the Langford model from FWHM GPFs. Heat-treated sample, 5 s at 600°C.

**Figure 14:**
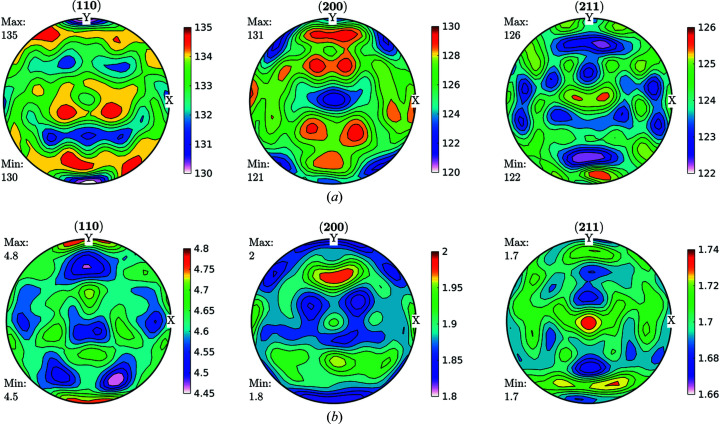
Recalculated GPFs for (*a*) domain size (nm) and (*b*) dislocation density (10^14^ m^−2^), as separated by following the Langford model from FWHM GPFs. Heat-treated sample, 5 s at 730°C.

For the heat-treated samples, the corresponding GPFs and the same GDF sections are shown in Figs. 10 ▸ and 11 ▸ for the treatment at 400°C, Figs. 12 ▸ and 13 ▸ at 600°C, and Figs. 14 ▸ and 15 ▸ at 730°C.

Fig. 16 ▸ shows the ODF for the 5 s, 730°C heat-treated sample (φ_2_ = 0° and φ_2_ = 45°). We observed that rotated-cube and α components became some of the weakest orientations, while the γ-fibre is the strongest. The rotated-cube and α components were composed of large grains with a very low dislocation content, and as a result are more prone to being consumed by the nucleation and further growth of those nuclei inside γ-fibre grains. Note that the increase in intensity of the γ-fibre for the 730°C heat-treated sample, with respect to the cold-rolled sample, is still far from optimum, probably due to the non-optimum time and temperature for the last annealing. Optimization of the process was not the purpose of the current research.

### 4.3. EBSD analysis

EBSD maps were measured using an FEG-SEM FEI *QUANTA* 200E with the acquisition software *TSL OIM DC 5* and data analysis was performed using *TSL OIM* (version 7.3; EDAX, Draper, UT, USA). Samples were polished with diamond paste of 9 µm, 6 µm, 3 µm and 1 µm, in that order. Then a final step of polishing using 0.05 µm colloidal silica was performed. The measurements were made from the TD direction, with RD parallel to the *x* axis and ND parallel to the *y* axis in 100 nm steps. No clean-up routine was employed prior to the microstructural analysis, and pixels were assigned to different grains if their misorientations were larger than 5°. The density of geometrically necessary dislocations (GNDs) was calculated using the method of Pantleon (2008 ▸) with a threshold misorientation of 5°.

Since the goal was to compare the microstructure of the material as a function of orientation, the information for each map was stored in different partitions. Considering the texture of the steel had two main components, the α-fibre and γ-fibre, for each EBSD map, three partitions were created: one that is consistent in all measurements with crystal directions (011) parallel to RD, one with crystal directions (111) parallel to ND and the third one with all the remaining orientations, as shown in Fig. 17 ▸. The range in which an orientation was assigned to a component was up to 15° misorientation with the corresponding direction. For each partition, the average GND was calculated as well as the average grain size, using the average intercept length method, in both RD and ND directions.

The same analysis was performed for a cold-rolled sample (R75) and for three annealed samples for 5 s at 400°C (R75A400), 600°C (R75A600) and 730°C (R75A730).

Fig. 18 ▸ shows the evolution of dislocation densities for the cold-rolled sample and the three annealed samples. For the cold-rolled sample R75, it is clear that the γ-fibre stored more dislocations than the α-fibre which is consistent with the results obtained by Langford’s method. Also, the ρ_GND_ values are on the same order of those obtained by XRD.

**Figure 15:**
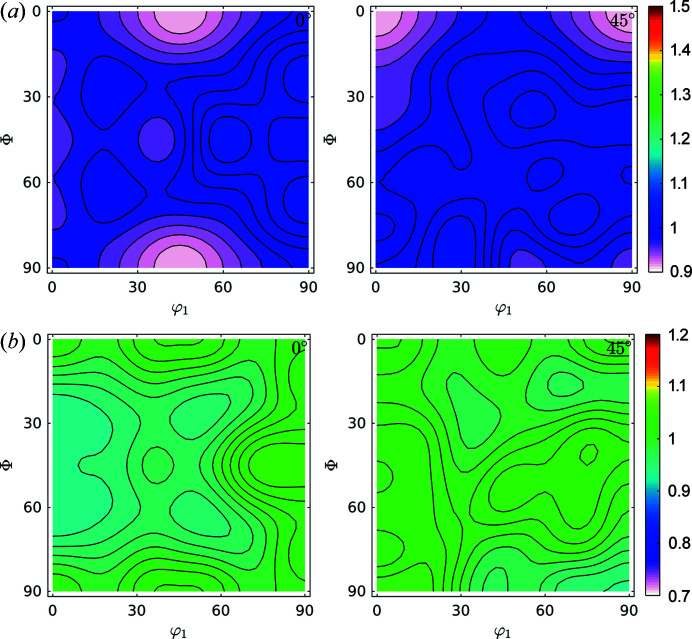
GDFs for (*a*) domain size and (*b*) dislocation density at φ_2_ = 0° and φ_2_ = 45°. Heat-treated sample, 5 s at 730°C.

The α-partition contains fewer dislocations than the γ-partition with intermediate values for the No α/γ-partition. The same trends observed for the R75 sample were also present for the R75A400 and R75A600 samples, with roughly the same values of GND for all partitions. Heat treatments at 400°C and 600°C are only enough to remove loosely stored dislocations and not compact arrays. For the R75A730 sample, dislocation density dropped drastically to a value on the order of 0.5 × 10^14^ m^−2^ for all partitions. This means that for annealing temperatures of 730°C most dislocations introduced by deformation were cleaned either by recombination or sub-grain and grain boundary migration. Further, for the R75A730 sample, all partitions have the same values of GND, which is also consistent with the information obtained from Langford’s method (Fig. 15 ▸) where it was shown that the domain size and strain distribution lost all correlation with texture.

Regarding domain sizes, measured as intercept lengths along ND and RD, it can be seen from Figs. 19 ▸ and 20 ▸ that the α-partition has a larger average size than the γ-partition, along both ND and RD, for the rolled sample. The No α/γ-partition, for all other components, has lower grain sizes than the γ- and α-partitions.

In addition, there are no great variations in the grain size for the samples annealed at 400°C and 600°C. Only loose dis­location arrays, those that cannot be detected by EBSD, are annealed at the two temperatures. For the sample annealed at 730°C a clear rise in the average grain size can be observed, as well as the loss of correlation between texture and grain size, which is also consistent with the GND and the Langford analysis. In fact, the larger abundance of GNDs present in the γ-fibre, once annealed, will produce grain sizes on the component that are larger than in the α-fibre.

## 5. Discussion

Current capabilities of high-energy synchrotron experiments and analysis allow us to realize, for the first time, a whole calculation of defect contents as a function of crystalline orientations, what is formally known as generalized orientation distribution functions, GDF. The requirements to achieve such analysis are, at present, satisfied by the angular accuracy, spatial resolution of image plate detectors, brilliance of current synchrotron radiation sources, *etc.*

Regarding the current models for separating the contributions stemming from different physical phenomena – like domain sizes, dislocations, twinning, *etc.* – they are still behind present needs. Currently, there are two approaches to obtain quantitative information from peak broadening: the analytical methods, which involve fitting analytical functions to the diffraction profiles; and the Fourier methods, which model the microstructure of the material and then fit the Fourier coefficients of the theoretical functions with those obtained from the measured profiles.

The analytical methods are easier to implement, and allow separation of the contributions of the different kinds of defects, relating such defects with the shape of the profile, in our case, Lorentzian for size contributions and Gaussian for strain contributions. However, such separation is only an approximation since it is known that, for compact arrays of dislocations, the strain contribution to peak broadening is also Lorentzian-like, which means that the length *L* ^*hkl*^ in equations (1) and (5) should be interpreted as the minimum length between the coherent diffracting crystallites in the *hkl* direction and the distance between arrays of dislocations. The high brilliance of the X-ray beam allows us to reasonably assume that the latter definition is more accurate. Further, the simplification of using the Langford model is useful for separating domain sizes and dislocation densities in materials like those presented in this work, in which the influence of other defects, like twinning, is negligible. Nevertheless, the methods based on the separation of defect contributions by analytical functions are biased, and this is why we chose to validate its predictions using EBSD, another technique that enables us to explore the microstructure of a material in an independent and more direct way.

The Fourier methods, on the other hand, are not biased, since they model the physical microstructure of the material and then look at the resulting theoretical profile to compare it with the experimental one. These methods are excellent in analyzing the microstructure of materials where the texture is negligible, in which case it is completely reasonable to assume that the anisotropic broadening of the diffraction peaks can be attributed to variations of the average contrast factors 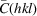 , which in turn depend solely on the sample direction for a given set of crystalline planes {*hkl*}. This means that both dislocation density and domain sizes will depend only on the sample direction, and its respective distributions should come from the contributions of all the diffraction peaks present in a given sample direction, all of which involve contributions of different crystal populations. This model becomes inaccurate when it is known that crystallographic texture affects the storage of defects, like in the study performed in the current work. A number of authors (Jóni *et al.*, 2013 ▸; Ungár *et al.*, 2015 ▸) have partially solved this limitation by analyzing diffraction peaks that originate from exactly the same population, allowing us to observe some correlations between crystallographic texture and defect storage. It could be said that those works partially map the GDF of dislocation density, since the kind of analysis presented can only be applied to a limited volume of the orientation space, which is the part in which the diffraction peaks come only from the crystals corresponding to the texture component of interest.

For mapping the dislocation density GDF, the diffraction profiles of many more sample orientations must be studied, which makes the analytical method based on the Langford model, although biased, the only one suitable for the task. It is the only method that allows separation of the size and strain contributions from single peak analysis with reasonable accuracy. From the GPF obtained via Langford, we obtain the GDF of defects based on the work of Rajmohan *et al.* (1997 ▸), Bunge (1982 ▸), Wcislak & Bunge (1996 ▸) and others (Ryo & Ryo, 2016 ▸; Miller *et al.*, 2005 ▸). The basis of our reasoning is that, if such GDF exists, it must be related to an ODF through an equation analogous to equation (8), which means that such distribution can be obtained by the same methods employed to obtain the ODF from the intensity pole figures. The existence of such function for the dislocation density seems reasonable from the work of Rajmohan, and from the fact that dislocation density behaves in the same way as the square of the FWHM of the diffraction peaks. We also chose to heuristically use the GPFs of size as input for the same analysis and evaluate its reasonableness from the consistency of the PF reconstruction from the calculated GDF, as well as the consistency with the EBSD measurements and the previous knowledge gathered for this steel. We also evaluated the dislocation density GDF with the same criteria. The worst scenario would be that we may not be delimiting correctly the dislocations assigned to the buildup of new domain borders and those kept, more or less, as loose dislocations contributing to dislocation density calculation.

**Figure 16:**
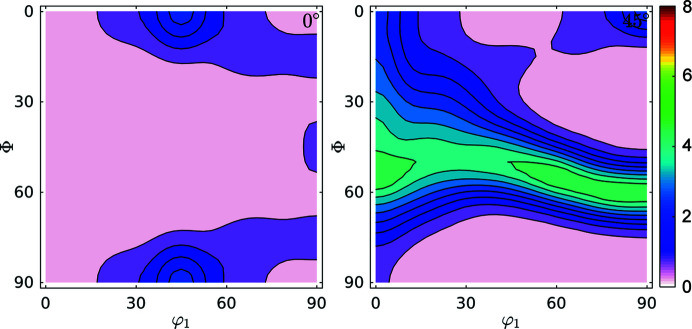
ODF for the 5 s, 730°C heat-treated sample. φ_2_ = 0° and φ_2_ = 45°.

**Figure 17:**
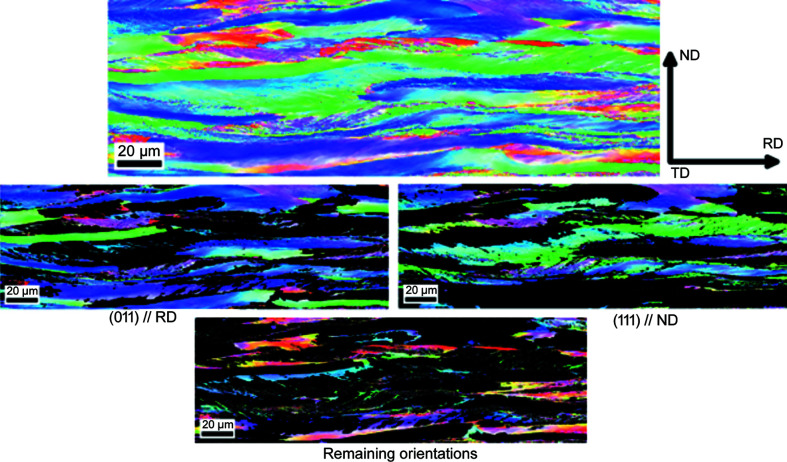
EBSD maps for the cold-rolled sample R75, for the partitions created to analyse the microstructure as a function of texture. For verifying the results obtained from XRD, three partitions were created: orientations along the α-fibre (011) // RD, orientations along the γ-fibre (111) // ND and all remaining orientations.

**Figure 18:**
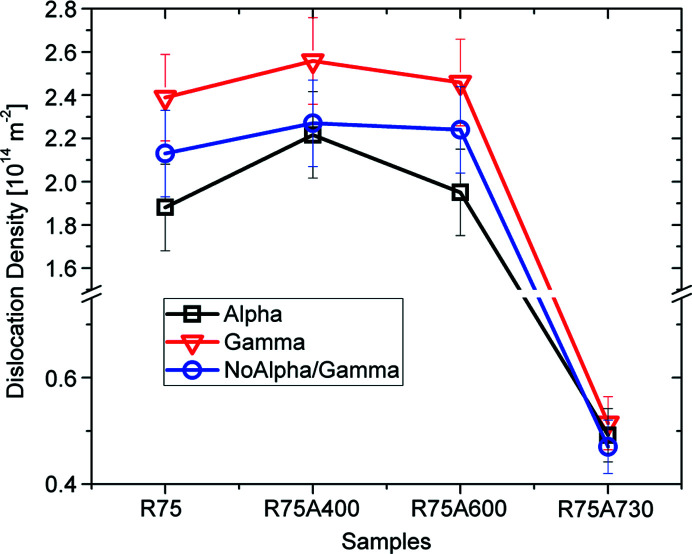
Dislocation densities for the cold-rolled and annealed samples. EBSD results show that the γ-fibre accumulates a greater number of dislocations for both the cold-rolled sample and the samples annealed at 400°C and 600°C. For the sample annealed at 730°C, the density of dislocations becomes homogeneous for all orientations. This is in agreement with the observations made from the Langford analysis from XRD.

**Figure 19:**
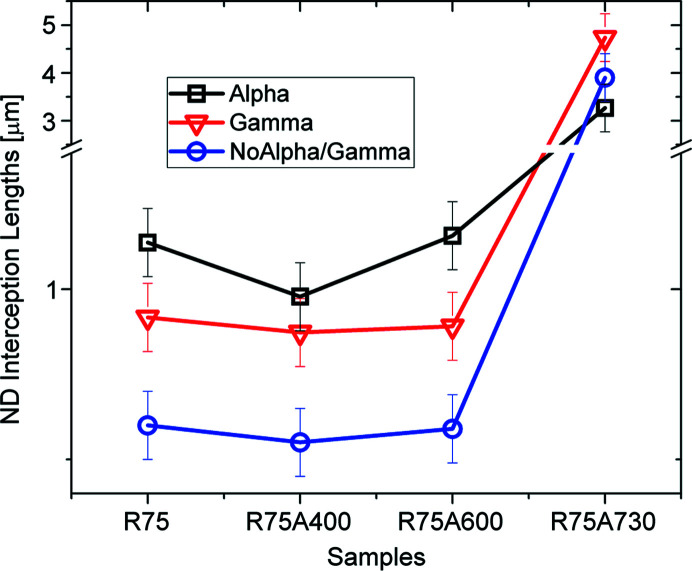
Average intercept lengths along the ND direction for each partition and each sample. For grain size, the α-fibre grows larger grains in both the cold-rolled sample and the samples annealed at 400°C and 600°C. Also, the grain size becomes quite homogeneous for the sample annealed at 730°C. All these results are also in agreement with those obtained from XRD analysis.

Figs. 3 ▸–13 ▸ show a strong relationship between the broadening, both in amount and in shape, of certain crystallographic directions and the corresponding crystallographic orientations, which suggest that the broadening observed in, say the (110) GPF, will be observed in the (200) GPF in a way consistent with the texture components measured in the ODF [*i.e.* if the poles corresponding to the γ-fibre in the (110) GPF show a larger broadening, the poles of the same fibre but in the (200) GPF should also be larger], which is precisely the observed behaviour. What is more, the crystal symmetry is imposed in all the GPFs, as is to be expected from equation (8). This behaviour further suggests that those GPFs are projections of a GDF, and can thus be processed by a pole figure inversion method to obtain a GDF linking crystalline orientations and features of the microstructure such as dis­location density and domain size, provided that the effect of the contrast factors does not destroy the correlation observed in the GPFs. These assumptions are equivalent with those made in Langford’s model, *i.e.* that microstructure is firmly correlated with crystalline orientation. The correlation between texture and defect storage is not observable in the annealed sample, as seen in Figs. 14–16 ▸ ▸ ▸, which is expected given the known behaviour of IF steels, and it will be addressed later.

Comparison between experimental and recalculated PFs is a known and proven way to check the quality of the data obtained during PF measurement. We can see that the recalculated GPFs obtained from the pole figure inversion algorithm show good agreement with the experimental GPFs. This is true for the FWHM, size and dislocation density GPFs. Furthermore, the quality of the reconstruction remains the same when the data obtained from the low intensity peaks are removed. This also addresses and removes one common criticism made to the line profile analysis, namely the fact that low-intensity peaks always tend to be broader than high-intensity peaks.

It is known that rolled IF steels tend to store dislocations mainly in the γ-fibre, which helps to enhance the drawability of steel sheets after proper recrystallization procedure. In fact, Every & Hatherly (1974 ▸) showed the following hierarchy for the stored energy as a function of fibre orientation for highly deformed steels,

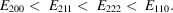

This hierarchy has never been contradicted by recent measurements, even though published numerical values are relatively scattered (Borbély *et al.*, 2000 ▸; Castelnau *et al.*, 2001 ▸; Rajmohan *et al.*, 1997*a* ▸; Wauthier-Monnin *et al.*, 2015 ▸). Moreover, it is in complete agreement with the results shown in this work using the Langford approach.

It is also known that the dislocation arrays formed during annealing can only be removed at temperatures higher than 700°C. The GDF for dislocation densities in Fig. 10 ▸ shows precisely this behaviour, *i.e.* φ_2_ = 45° sections of the GDF show clearly how the orientations along the γ-fibre have the highest number of dislocations for all orientations, while the crystals orientated along the α-fibre and in the rotated cube component have the minimum number of dislocations. It also shows that all other orientations have an intermediate number of dislocations. Size-GDF indicates that the components with more dislocations also have smaller domains sizes, and that the components with fewer dislocations have larger domain sizes.

It can be said that assuming Langford’s model is valid has important consequences when determining the microstructure: the dislocations considered by the model are those contained in loose arrays and the domain sizes are perhaps smaller than expected, because compact dislocation arrays are interpreted as domain boundaries. Moreover, on the current experimental setup, the high brilliance of the synchrotron beam may enforce the detection of dislocation arrays as if they were domain boundaries, decreasing the domain size even more. That said, there still remains the question of whether the smaller domains can be attributed to smaller grains or to higher amounts of piled up dislocations, which also produce a Lorentzian broadening that cannot be separated from the pure size contribution to peak broadening. The correct limit in between which dislocations are considered as such and considered as domain limits remains somewhat murky, but we expect none of them stay undetected but rather are assigned to one or the other set of dislocations. The discussion may become Byzantine in some aspects, since the limit between both perhaps artificially separated dislocation accumulations is highly dependent on the measurement method itself (*e.g.* synchrotron brilliance).

Figs. 12 ▸ and 14 ▸ show that the trends observed for the cold-rolled sample remain for annealing temperatures of 400°C and 600°C, and that in the annealing performed at 730°C only the γ-fibre remains, and that dislocation density and crystallite size become more or less homogeneous for all orientations, according to what is known for this material.

Furthermore, since the correlation between orientation and microstructure was so clear, it was possible to analyze EBSD maps for the same sheets and study the microstructure of this material on a different scale, comparing grain sizes along different directions and the density of GNDs for all four samples. Again, it was shown that crystals along the γ-fibre have smaller grains and higher GNDs than the crystals belonging to the α-fibre and the rotated cube component, and that all the remaining orientations store an intermediate number of defects. EBSD measurements confirm this behaviour for the samples annealed at 400°C and 600°C, and the microstructure becomes homogeneous with all orientations having a similar number of GNDs and grain size for the sample annealed at 730°C.

To summarize, based on the assumption that dislocation density depends on the crystal orientation in a way that can be represented by a GODF, we obtain size and dislocation density GPFs, based on the Langford model, which was the only one that allowed us to separate such contributions when performing single peak analysis. We found that GDFs obtained from such GPFs are self-consistent in the sense that the GDFs obtained produce recalculated GPFs which are similar to the measured ones. Additionally, the current results have been obtained in a system, rolled IF steel, where much of the physics and metallurgy has been explored in the past, and we found that our results are consistent with what is known for this material. Finally, we performed further confirmation of the results by EBSD analysis. Guided by the GDF, we looked for the correct partitions in the EBSD maps and confirmed the persistence of certain anisotropic accumulation processes despite the uncertainty of the kind and quantity of dislocations and type of arrays, character of domain boundaries, *etc*. It seems clear that, at least in the current system, certain invariance on the scale of analysis can become fruitful for the understanding of defect accumulation. In the first approximation, the accumulation of defects is scale invariant, *i.e.* no matter which method is used and no matter the resolution scale, the sizes and dislocation accumulations correlate at those different scales. A further search in different systems is underway to confirm that trend.

As for the dependence of the crystallite size on the sample orientation, the experience from EBSD and metallographic grain-size measurements show that the shape of grains is mostly determined by the sample symmetry of deformation processes, rather than by their crystal orientation, which favours the results given by the homogeneous approach. At this point it is worth remembering that the grain definition from metallography and EBSD, coming from boundaries drawn looking for rather large misorientations (≳5°) between neighbouring crystals, is conceptually different from the crystallite size distribution that is constructed from the sizes of simply connected coherent diffraction domains. For synchrotron radiation this means that two crystals with a mis­orientation larger than the beam divergence (≳0.01°) will make different contributions to the size distribution. In this context, it is no longer obvious that what is known from metallography and EBSD will also hold true for X-ray size distributions. A consequence of this is that it is not necessarily true that the crystallite size should depend only on the sample direction, but also on the crystallographic orientation.

Note that the average value of dislocation density given by both Langford decomposition and EBSD is ∼10^14^ m^−2^, which means that both methods are equally reliable if one is only interested in estimating the order of magnitude of the dis­location density of the sample as a whole, instead on the details of their distribution. Moreover, the proportional distribution of defects with respect to orientation components is information that remains valid despite the fact that absolute values are unknown.

## 6. Conclusions

Cold-rolled IF steel was analyzed by XRD using synchrotron light. The material was chosen because of its importance in many industrial processes and because the development of its microstructure with rolling and annealing is well known. Synchrotron light was chosen because of high intensity, high brilliance and the allowed low-divergence angle, making high-resolution measurements of crystallographic texture and microstructure of the material possible, by relating the intensity and broadening of the diffraction peaks with the sample orientation.

The broadening of the peaks was analyzed using the Langford model for line profile analysis. Langford’s method, while biased and more primitive at first sight, can model the main features of the microstructure of textured materials with reasonable accuracy, and can accomplish this by performing a single peak analysis.

This method was used to construct GPFs for defects, and such PFs were used to calculate the corresponding GDFs using standard pole-figure-to-ODF inversion algorithms. The calculated GDFs were consistent with the measured GPF, and the conclusions drawn from such GDFs were also consistent with what is known for IF steels subject to cold-rolling and annealing processes.

The results obtained with XRD were also compared using EBSD, another well established and independent technique for analyzing the microstructure of materials on a different scale. The EBSD maps were partitioned in the three main components of texture, namely the α-fibre, the γ-fibre and one additional partition consisting of all the remaining orientations. The partitions were created to check the predictions made using the GDFs of size and dislocation density. The defect storage of every partition was estimated by means of the average intercept length of grains and the average of the GNDs, and we observed that grains belonging to the γ-fibre tend to store more defects than those belonging to the α-fibre, and that the remaining orientations lie more or less in the middle. All these results were in perfect agreement with those obtained by applying Langford’s method to XRD measurements, which in turn were in good agreement with the previous experiments for this material.

The same experiments were repeated for annealed samples at 400°C, 600°C and 730°C, and we observed that the samples annealed at 400°C and 600°C show more or less the same microstructure than the cold-rolled sample, according to what is known for this steel. We also observed that the annealing at 730°C removes the majority of the dislocations of the material, homogenizing dislocation with respect to orientation, which was also in agreement with what is known for rolled IF steels. More importantly, the same results were obtained from both EBSD and XRD measurements when Langford’s method and GDF were applied.

The analysis described in this paper demonstrates how peak broadening is related to sample orientation in correlation with texture. This correlation allows us to construct GODFs from GPFs obtained by means of XRD, and perform an analysis of peak broadening according to Langford’s model. These GDFs succeeded in describing, albeit in a qualitative manner, how defects are stored in different texture components, which means that texture does not only command the macroscopic anisotropy of crystalline materials but also defines the anisotropy of defect storage.

A final word is owed to the caveat that may be interceded regarding the impossibility of knowing the character of the functional relationship between GDFs and GPFs in the sense of our ignorance of what would happen with the defect content on a hypothetical crystal oriented in such a way that it cannot be detected experimentally. The question arises of whether the defect content can be reconstructed from the information given on the orientations that contain a reasonable number of crystals. The answer is clearly no, but the concern becomes, in our opinion, immaterial since the reconstruction appears adequate and defines a relationship between both spaces that reveals a functional in terms of its univocally and reversible character.

We must emphasize that the calculation of full GDFs has been realized for the first time, after many years of being suggested as a method for anisotropic structure and microstructure analysis. It may become a customary method for such analysis in deformed and heat-treated metals and alloys. Even though these results are by no means conclusive, they are promising enough to justify its applications to more materials in order to verify the scope of this model.

## Figures and Tables

**Figure 11:**
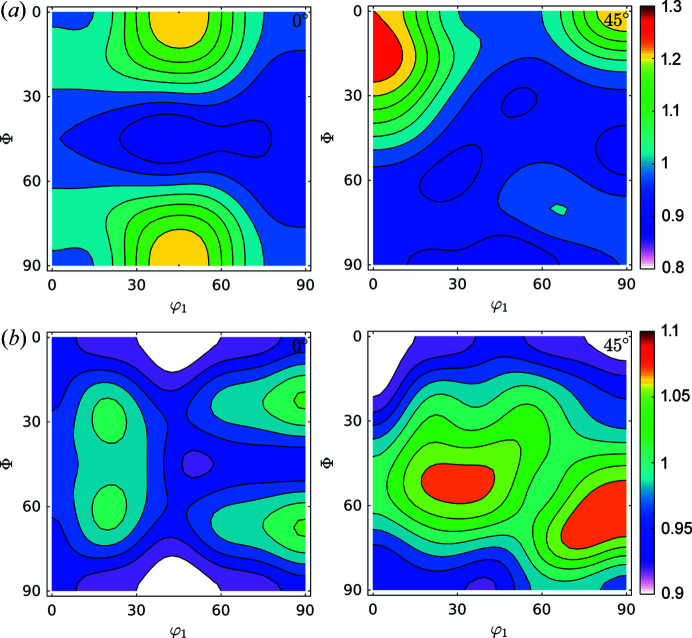
GDFs for (*a*) domain size and (*b*) dislocation density at φ_2_ = 0° and φ_2_ = 45°. Heat-treated sample, 5 s at 400°C.

**Figure 13:**
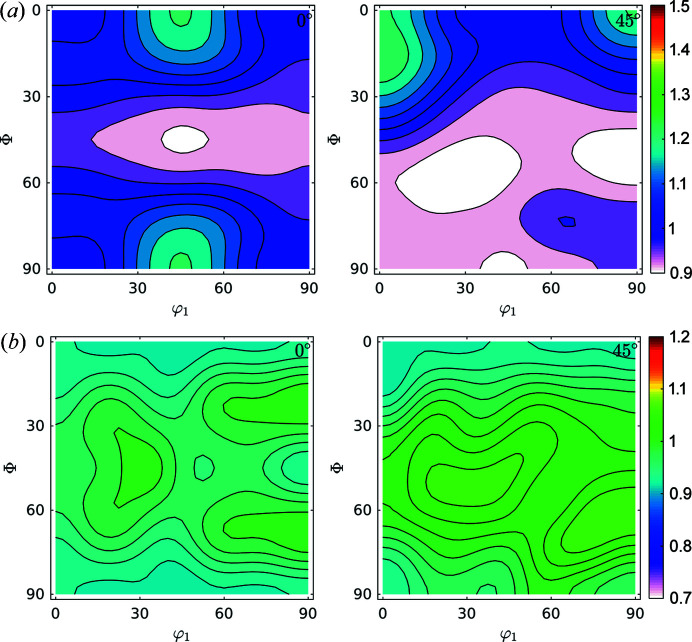
GDFs for (*a*) domain size and (*b*) dislocation density at φ_2_ = 0° and φ_2_ = 45°. Heat-treated sample, 5 s at 600°C.

**Figure 20:**
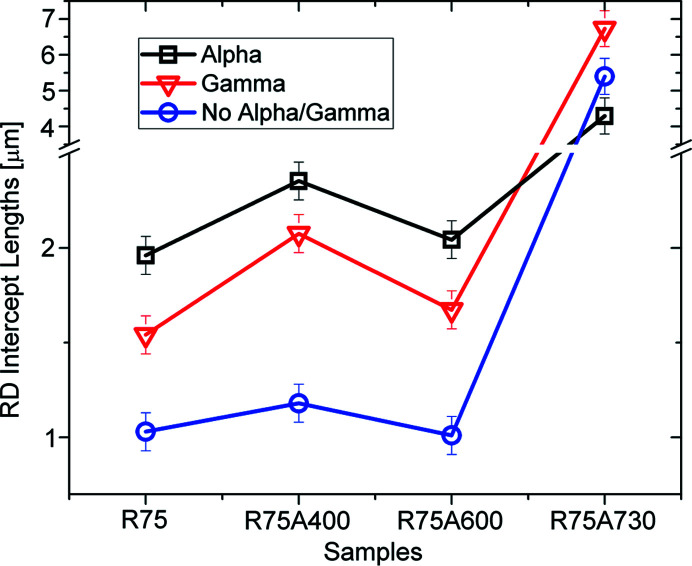
Average intercept lengths along the RD direction, for each partition and sample, further confirm the trend observed in Fig. 19 ▸.

## Footnotes

1 There are libraries that allow the exact calculation of the Voigt profile [*e.g.* Johnson & Wuttke: libcerf, numeric library for complex error functions (version 1.17), http://apps.jcns.fz-juelich.de/libcerf, or Limandri *et al.* (2008 ▸)]. However, there are still programs widely used that rely on the pseudo-Voigt calculations, *e.g.* the *FULLPROF* suite (Rodríguez-Carvajal, 1993 ▸; Roisnel & Rodriguez-Carvajal, 2000 ▸; Rodriguez-Carvajal & Roisnel, 1998 ▸), in order to implement the same algorithm in our calculations.
